# An Eye in the Palm of Your Hand: Alterations in Visual Processing Near the Hand, a Mini-Review

**DOI:** 10.3389/fncom.2016.00037

**Published:** 2016-04-18

**Authors:** Carolyn J. Perry, Prakash Amarasooriya, Mazyar Fallah

**Affiliations:** ^1^Visual Perception and Attention Laboratory, York UniversityToronto, ON, Canada; ^2^Centre for Vision Research, York UniversityToronto, ON, Canada; ^3^School of Kinesiology and Health Science, York UniversityToronto, ON, Canada; ^4^Canadian Action and Perception Network, York UniversityToronto, ON, Canada

**Keywords:** attention, vision, sensorimotor integration, reaching and grasping, peripersonal space

## Abstract

Feedback within the oculomotor system improves visual processing at eye movement end points, also termed a visual grasp. We do not just view the world around us however, we also reach out and grab things with our hands. A growing body of literature suggests that visual processing in near-hand space is altered. The control systems for moving either the eyes or the hands rely on parallel networks of fronto-parietal regions, which have feedback connections to visual areas. Since the oculomotor system effects on visual processing occur through feedback, both through the motor plan and the motor efference copy, a parallel system where reaching and/or grasping motor-related activity also affects visual processing is likely. Areas in the posterior parietal cortex, for example, receive proprioceptive and visual information used to guide actions, as well as motor efference signals. This trio of information channels is all that would be necessary to produce spatial allocation of reach-related visual attention. We review evidence from behavioral and neurophysiological studies that support the hypothesis that feedback from the reaching and/or grasping motor control networks affects visual processing while noting ways in which it differs from that seen within the oculomotor system. We also suggest that object affordances may represent the neural mechanism through which certain object features are selected for preferential processing when stimuli are near the hand. Finally, we summarize the two effector-based feedback systems and discuss how having separate but parallel effector systems allows for efficient decoupling of eye and hand movements.

## Introduction

Accumulating behavioral evidence has shown that visual processing is altered near the hand. Speeded target detection and figure-ground assignment (Reed et al., 2006, 2010; Jackson et al., 2010), improvements in working memory (Tseng and Bridgeman, 2011), orientation processing (Craighero et al., 1999; Bekkering and Neggers, 2002; Hannus et al., 2005; Gutteling et al., 2011, 2013), target discrimination (Deubel et al., 1998), and in reaching and grasping precision (Brown et al., 2008), are just some of the effects seen when a reach places a hand near a visual stimulus. In addition, these alterations are seen whether the hand is nearby due to a sustained reach or if the hand is moved towards the visual stimulus during each trial in a more active manner. What remains a topic of debate is the mechanism by which these alterations in visual processing occur. A number of studies suggest that visual processing near the hand is altered through spatial attention selection mechanisms (di Pellegrino and Frassinetti, 2000; Schendel and Robertson, 2004; Reed et al., 2006, 2010; Abrams et al., 2008). These studies have hypothesized that populations of fronto-parietal bimodal neurons underlie enhanced visual selection in near-hand space; however, these neurons are also thought to influence near-hand processing in the absence of spatial attention influences (Brown et al., 2008). More recently, enhanced magnocellular processing has been postulated as an alternative explanation for the near-hand effect (Gozli et al., 2012). For this review, we investigate the hypothesis that these effects are driven by a novel, effector specific, attentional selection mechanism that is different from either oculomotor-driven visual spatial or feature-based attention, and is mediated by feedback from fronto-parietal regions involved in reaching and grasping networks. We will first review the anatomical similarities between the oculomotor and the reaching/grasping networks, and provide evidence of feedback influences within the oculomotor system. We will then compare the neurophysiological alterations in visual processing near the hand to alterations in visual processing due to the oculomotor system and provide supporting evidence of feedback influences in the reaching and grasping system. We suggest that links between the visual system and the motor systems could drive enhanced processing of action-relevant object features, but that de-coupled eye and hand movements indicate the need for separate, effector-based selection mechanisms.

## Neural Circuitry

**Figure 1:**
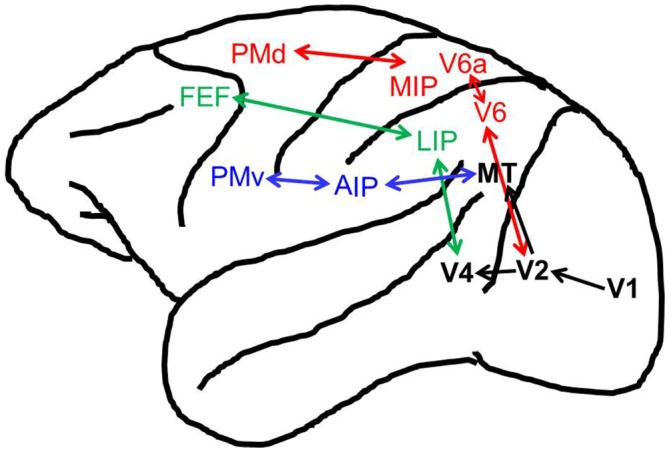
**Reach, grasp, and oculomotor control brain regions in the macaque.** Shown are the cortical brain regions associated with the reach (in red), grasp (in blue), oculomotor (in green), and visual (in black) systems. Not pictured are anatomical cross-talk connections between the reaching and grasping networks (i.e., between V6A and anterior intraparietal (AIP)/Ventral premotor cortex (PMv), see Fattori et al., 2015).

The reaching, grasping, and oculomotor systems all involve parallel networks of fronto-parietal areas (Figure 1). A dorsomedial stream, projecting from visual area V6 (Rizzolatti and Matelli, 2003; Passarelli et al., 2011), consisting of the medial intraparietal (MIP) area and area V6A in the superior parietal lobule (SPL), along with the dorsal premotor cortex (PMd) in the frontal lobe, which forms what is thought to be the neural network for reaching in the non-human primate (Caminiti et al., 1996; Culham et al., 2006; Filimon, 2010), with homologs in humans (Culham et al., 2006; Filimon, 2010). As with reaching, it has been suggested that there is a parallel dorsolateral circuit specialized for grasping (Fagg and Arbib, 1998; Luppino et al., 2001; Filimon, 2010) that projects from visual area MT/V5 (Rizzolatti and Matelli, 2003), and that this circuit is mainly dependent upon connections between the anterior intraparietal (AIP) region in the inferior parietal lobule (IPL) and the ventral premotor cortex (PMv), with homologous areas in humans (Fagg and Arbib, 1998; Culham et al., 2003, 2006; Frey et al., 2005). The reaching and grasping circuits however, appear to not be as completely functionally distinct as once thought as recent work has also found grasping related activity in the dorsomedial stream in non-human primate (Raos et al., 2003, 2004; Fattori et al., 2009, 2010, 2012) and human populations (Gallivan et al., 2011; Monaco et al., 2011). In fact, it has been suggested that the visual, somatosensory, and motor properties of V6A indicate a role for this area in the online error control for all of prehension, including reaching and grasping (Fattori et al., 2015). For movements of the eyes, the cortical oculomotor system in non-human primates and humans is comprised of the lateral intrapariental area (LIP)/parietal eye fields (PEF) and the frontal eye fields (FEF; Goldberg and Segraves, 1989; Bisley and Goldberg, 2003; Culham and Valyear, 2006; Culham et al., 2006). Due to the similarity between the anatomical components of these systems, we suggest that it is possible that oculomotor feedback mechanisms enhancing visual processing, could be replicated by the reaching and grasping networks to alter visual processing near the hand.

## Feedback in the Oculomotor System

The influence of feedback, from fronto-parietal motor related areas, on visual processing is already well-supported for the oculomotor system. Early psychophysical work established an indirect link between alterations in visual processing due to shifts in attention and saccade motor planning (Rizzolatti et al., 1987; Kowler et al., 1995; Sheliga et al., 1994; Deubel and Schneider, 1996; Kustov and Robinson, 1996; Nobre et al., 2000; Castet and Montagnini, 2006; van der Stigchel and Theeuwes, 2006; Baldauf and Deubel, 2008). In general, visual processing was improved when a visual target coincided with the endpoint of a planned saccade suggesting a close relationship between the oculomotor system and attention related changes in visual processing. These studies led to investigations that more causally associated activations of eye-movement related brain regions to shifts in spatial attention and consequently alterations in visual processing at the end points of planned saccades (Moore and Fallah, 2001, 2004; Moore and Armstrong, 2003; Müller et al., 2005; Neggers et al., 2007; Van Ettinger-Veenstra et al., 2009; Gutteling et al., 2010; Bosch et al., 2013). For example, subthreshold microstimulation of the FEF resulted in increased visual sensitivity at the end-point of the unactivated motor plan behaviorally (Moore and Fallah, 2001, 2004) and within area V4 (Moore and Armstrong, 2003). This would suggest that recurrent connections between FEF and V4 allow for signals from FEF to feed back into the occipital lobe to influence subsequent visual processing (Armstrong et al., 2006; Armstrong and Moore, 2007; Ekstrom et al., 2008, 2009; Squire et al., 2012). Further evidence in primates comes from a study by Supèr et al. (2004) who found that in primary visual cortex neural activity corresponding to the location of the saccade target was enhanced approximately 100 ms before the onset of memory and visually-guided saccades. Studies in humans using transcranial magnetic stimulation (TMS) provide additional support for oculomotor feedback modulating visual processing. A single TMS pulse activates neurons in the targeted area. As such single pulse TMS over FEF enhances visual processing (Grosbras and Paus, 2003; Ruff et al., 2008; Van Ettinger-Veenstra et al., 2009) presumably by activating the feedback connections to visual processing areas. In contrast, a triple pulse disrupts the normal processing in an area. Triple pulse TMS used to disrupt the FEF results in impaired discrimination of a subsequently presented target (Neggers et al., 2007) suggesting that oculomotor feedback is necessary for spatial attention. Both the primate microstimulation studies and the human TMS studies support oculomotor feedback producing spatial attention effects behaviorally and within visual neurons. This would require attention signals to occur in the frontal lobe and propagate back to the occipital lobe. This is indeed what Van Ettinger-Veenstra et al. (2009) showed with EEG neuroimaging. They found that frontal activity associated with a saccade-go signal preceded activity in the occipital cortex associated with the appearance of a visual target. Thus, feedback projections from oculomotor-related frontal areas alter processing in posteriorly located visual areas.

## Visual Processing Near the Hand

As mentioned previously, behavioral studies have provided indirect evidence suggesting that the space near the hand is prioritized. One prevailing theory suggests that alterations in visual processing occur as a result of attentional selection of near-hand space (di Pellegrino and Frassinetti, 2000; Schendel and Robertson, 2004; Reed et al., 2006, 2010; Abrams et al., 2008; Brown et al., 2008). Much like visual processing at the end point of a saccade is altered, the parallel within the reaching and grasping system would be a change in visual processing that occurs at the end point of a reach or grasp, i.e., in the workspace near the hand. One can imagine the benefit of this type of mechanism. This is especially true when reaching for an object while simultaneously viewing something in a different location that draws oculomotor driven spatial attention away from the object to be picked up. The underlying neural mechanisms that would drive altered visual processing near the hand have, as yet, not been well studied. A very recent neurophysiological study however, has shed light on the neural underpinnings of near-hand visual processing (Perry et al., 2015). Neuronal activity was recorded from area V2 which is an area that is known to be selective for orientation (Motter, 1993), a feature important for reaching and grasping (Murata et al., 2000; Raos et al., 2004; Fattori et al., 2009), modulated by attention (Motter, 1993; Luck et al., 1997), and directly linked to fronto-parietal reaching and grasping areas (Gattass et al., 1997; Passarelli et al., 2011; Fattori et al., 2015). Instead of allocating classic visual spatial attention with a cue (Moran and Desimone, 1985; Motter, 1993; McAdams and Maunsell, 1999; Treue and Martinez-Trujillo, 1999), Perry et al. (2015) used the presence or absence of a nearby hand to determine the effects of near-hand attention on neuronal responses in area V2. Under these conditions, there was a significant increase in response at the preferred orientation when the hand was nearby. This is consistent with classic visual spatial studies which produce a “gain-modulation” of neuronal responses: responses are multipled by the same factor regardless of selectivity (McAdams and Maunsell, 1999; Seidemann and Newsome, 1999; Treue and Martinez-Trujillo, 1999; McAdams and Reid, 2005). This results in a scaling of the tuning curve. However in contrast to gain modulation, there was no corresponding increase at the orthogonal orientation when the hand was near. Consequently, this produced a sharpening, instead of a scaling, of the orientation tuning curves when the hand was near, suggesting a different underlying mechanism than for oculomotor driven spatial attention. Sharpening of orientation tuning curves would result in greater orientation selectivity.

In addition to spatial attention, neuronal enhancement is also found with feature-based attention, where attending to a feature (such as a vertical bar) enhances processing of that specific feature (vertical), which aids greatly in visual search. Feature-based attention is described by the feature-similarity gain model of attention which predicts that enhancement of neuronal responses are strongest when the orientation of the grasp target (attended feature) and the orientation of the visual stimulus are matched, falling off as the difference in their orientations increased (Treue and Martinez-Trujillo, 1999). No such relationship was found. These results (Perry et al., 2015) suggest then that the attentional prioritization of near-hand space does not conform to known spatial or feature-based attentional mechanisms and that a novel, effector based, mechanism exists. This mechanism would preferentially process features (such as orientation) necessary for grasping, which would then improve the accuracy of an upcoming grasp.

## Evidence for Feedback in the Reaching and Grasping Systems

While the effects of near-hand attention are seen in early visual areas, behaviorally these effects cannot be driven by the oculomotor system. The control system for near-hand attention, albeit separate from the oculomotor system, would likely be driven through the parallel feedback from fronto-parietal motor planning areas. It has been shown that neuronal response variability is reduced in premotor cortex during reaching (Churchland et al., 2010) and in the FEF during oculomotor preparation (Purcell et al., 2012). Notably, neurons in V4 undergo a reduction in neuronal response variability prior to the onset of a saccade (Steinmetz and Moore, 2010). This suggests that reductions in oculomotor response variability propagate back to posteriorly located visual processing regions. If feedback from fronto-parietal reaching and grasping networks is the method through which neurons in V2 undergo alterations in their response properties (such as sharpened tuning—Perry et al., 2015), it would be expected that response variability would also be reduced. This is, in fact, what was found (Perry et al., 2015). Thus, both oculomotor and near-hand spatial attention rely on feedback projections which concomitantly reduce response variability.

In human populations, this premise of feedback connections mediating changes in visual response properties was tested by Gutteling et al. (2013). They investigated whether activation of the anterior portion of the intraparietal sulcus (aIPS) prior to a grasping or pointing movement improved orientation perception. aIPS has been shown to be part of a network of fronto-parietal areas that are involved in the control of grasping movements (Taira et al., 1990; Gallese et al., 1994; Sakata et al., 1995). Furthermore, aIPS has been shown to be selective for the orientation of the object to be grasped (Murata et al., 2000) and connected to occipital visual areas (Nakamura et al., 2001; Ruff et al., 2008; Blankenburg et al., 2010), including ventral stream regions (Borra et al., 2008) that would be sensitive to changes in orientation. Activation of aIPS during action preparation (Gutteling et al., 2013) improved orientation sensitivity, suggesting that aIPS is involved in modulating visual information during action planning. In addition, compared to pointing, grasping a 3-dimensional oriented bar, has been shown with electroencephalography to strengthen the N1 component and associated selection negativity in lateral occipital regions suggesting that the plan to grasp influences early ventral stream visual processing (orientation) of action-relevant features (Van Elk et al., 2010). Improved sensitivity and strengthened selection negativity is consistent with improved orientation tuning found in non-human primate V2 neurons when a hand is nearby (Perry et al., 2015).

Area V6A is another candidate area whose feedback could sharpen orientation tuning, as it has been found to be sensitive to the orientation of the wrist (Fattori et al., 2009), selective for grip type (Fattori et al., 2010), contains cells selective for orientation (Gamberini et al., 2011), and has direct connections to early visual processing areas (Passarelli et al., 2011). In addition, activity in V6A has been shown to be modulated by shifts in covert, oculomotor driven, spatial attention (Galletti et al., 2010), suggesting that it may play a similar role in hand driven attention.

Recurrent feedback loops between fronto-parietal and early visual processing areas (e.g., V2) would provide relevant corollary motor discharge information to enhance visual information relevant to reaching and grasping objects (i.e., sharpened orientation tuning) that would then update ongoing motor plans. As a movement progresses, sharpened orientation tuning information could be used to improve or correct hand shaping and wrist orientation resulting in improved reach and grasp accuracy. Given that V6A is thought to be involved in online error control of both reaching and grasping (Fattori et al., 2015), recurrent feedback loops between V2 and V6A are the likely candidate mechanism to underlie this process.

## Affordances

Orientation is considered to be part of the processing that occurs in the ventral stream that results in object recognition. It is not thought to be necessary for processes in the dorsal stream that culminate in knowing where something is, for computations of complex motion of an object, or for execution of movement. Why then would orientation processing in V2 be improved simply because the hand is near? Close links between the visual and motor systems have been at the core of the *affordance* literature for years. Gibson (1979) suggested that one of the key functions of the visual system was to provide information to the motor system about the possible actions that could be implemented, or alternatively, the possible actions that the visual information *affords*. Since then, Tucker and Ellis (1998, 2001) and Ellis and Tucker (2001) have argued that the motor system itself could extract visually pertinent information that would produce affordances. In fact, they have used the term micro-affordances to refer to object properties that are action-relevant and could be used to inform subsequent movements to interact with the object of interest (Tucker and Ellis, 2001). Orientation is an object feature that informs the “graspability” of an object. For example, object orientation can either facilitate or impede response times depending on whether the object orientation produces a motor affordance (Tucker and Ellis, 1998). In other words, the orientation of an object informs the grasp that needs to be planned. Regions within the parietal lobe, integral to reaching and grasping movements, show selectivity for the size, shape and orientation of an object both during fixation and grasping movements (Taira et al., 1990; Gallese et al., 1994; Murata et al., 2000; Fattori et al., 2009, 2010, 2012; Breveglieri et al., 2015), suggesting these areas play a key role in the integration of visual and motor information and object affordances. Therefore, orientation is a feature necessary to grasp objects accurately and is processed within the fronto-parietal grasping network, especially within area AIP.

Even if there is not a representation of the object as a whole in the dorsal stream, the vision for action theory (Goodale and Milner, 1992; Goodale, 2008, 2013) would also suggest that there are features of an object that are action relevant and therefore worthy of preferential processing, or attentional selection, by the dorsal stream action system. Patients with visual agnosia, who can still scale and orient their hand to an object to be grasped in spite of being unable to recognize the object they are grasping, speak to this point (Goodale et al., 1991, 1994; Milner et al., 2012). Given that object features such as orientation have been shown to affect subsequent motor affordances, and that object properties are extracted to inform the scale and orientation of the hand in patients who cannot recognize objects, it logically follows that orientation be an object feature preferentially processed within the dorsal stream in parallel to its processing within the ventral stream for object recognition.

## Advantages of Separate Effector Mechanisms

Being able to separate the deployment of attention between effectors allows for the decoupling of actions. Many examples exist of instances where we reach for one thing while looking elsewhere. In fact, optic ataxia, in which there is an inability to reach to peripheral targets, results from damage to the posterior parietal cortex (Milner and Goodale, 1995; Carey et al., 1997; Jackson et al., 2005). It has been shown that reaching to centrally located targets activates the MIP sulcus and PMd, while reaching to peripherally located targets additionally activates the parietal occipital junction and more rostral parts of PMd. These differentiated networks support dissociation between where gaze and grasp are deployed (Prado et al., 2005). Furthermore, recent work has shown that when a sequence of reaching movements are planned, visual discrimination is significantly enhanced not just at the first movement goal but also at the second (Baldauf et al., 2006; Baldauf and Deubel, 2008, 2009). So while an eye movement would be planned and then executed to the first target, the second is already enhanced suggesting that reach execution is separate from oculomotor planning and in turn, that movement planning and execution in the posterior parietal cortex already accommodates separate representations of gaze and reach targets (Jackson et al., 2009). These decoupled eye and hand movements are supported by the presence of neuronal populations in parietal areas that produce multiple types of reference frame transformations to encode targets in eye-centered or hand-/body-centered frames of reference (Lacquaniti et al., 1995; Batista et al., 1999, 2007; Buneo et al., 2002, 2008; Cohen and Andersen, 2002; Marzocchi et al., 2008; Chang et al., 2009; Chang and Snyder, 2010; McGuire and Sabes, 2011). As populations encoding targets in either eye- or hand-centered reference frames support decoupled movements, it follows then that there should exist separate effector-based attentional mechanisms.

## Conclusion

We have reviewed literature in support of the hypothesis that there exist parallel, but separate, effector-based attentional systems. Whereas the oculomotor system enhances visual responses through gain modulation, near-hand attention sharpens orientation tuning and, potentially, other features relevant to reaching and grasping. Thus, these effector-based systems may be specialized for the actions those effectors can perform. We suggest that improved orientation processing is a feature important for accurate reaching and grasping, and that separate effector-based attentional mechanisms allow for the decoupling of visual enhancements associated with eye and hand movements. Future investigations are needed to further support this hypothesis for example, by systematically testing grasp-relevant and irrelevant features. In addition, testing whether both the reaching and grasping or grasping alone is involved in near-hand attention which will provide details regarding which fronto-parietal networks may be involved and what other object features may be preferentially processed.

## Author Contributions

CJP, PA, and MF all contributed to the writing and revision of this article.

## Conflict of Interest Statement

The authors declare that the research was conducted in the absence of any commercial or financial relationships that could be construed as a potential conflict of interest.
